# Chitotriosidase: a biomarker of activity and severity in patients with sarcoidosis

**DOI:** 10.1186/s12931-019-1263-z

**Published:** 2020-01-06

**Authors:** David Bennett, Paolo Cameli, Nicola Lanzarone, Loredana Carobene, Nicola Bianchi, Annalisa Fui, Luigi Rizzi, Laura Bergantini, Giuseppe Cillis, D’ Alessandro Miriana, Maria Antonietta Mazzei, Rosa Metella Refini, Piersante Sestini, Elena Bargagli, Paola Rottoli

**Affiliations:** 1 Respiratory Diseases and Lung Transplantation Unit, Azienda Ospedaliera Universitaria Senese, Siena, Italy; 2 Department of Medical and Surgical Sciences & Neurosciences, University of Siena, Siena, Italy; 3 Internal Medicine Unit “C. Frugoni”, Centre for Rare Diseases, University Hospital of Bari, Bari, Italy; 4 Diagnostic Imaging Unit, Azienda Ospedaliera Universitaria Senese, Siena, Italy

**Keywords:** Chitotriosidase, Sarcoidosis, Biomarkers, Clinical evaluation

## Abstract

**Background:**

Serum chitotriosidase is a promising biomarker that has shown high specificity and sensitivity in patients with sarcoidosis. The aim of this study was to investigate correlations between serum chitotriosidase, clinical phenotypes, disease localizations and different radiological lung involvement and to identify clinical features associated with over-expression of chitotriosidase in a large cohort of sarcoidosis patients.

**Methods:**

Chitotriosidase activity was evaluated in a population of 694 consecutive patients (males 39%, age 55.8 ± 12.8 years). Clinical and respiratory functional characteristics, Clinical Outcome Scale (COS) classification, clinical phenotypes proposed by the GenPhenResA project, and radiological assessment, including CT scan, were collected. Serum sampling and clinical and functional assessments at follow-up were also included.

**Results:**

Significantly higher chitotriosidase activity was observed in sarcoidosis patients than in healthy controls (*p* < 0.0001). Evidence of lung fibrosis with reticular abnormalities and traction bronchiectasis at High resolution CT, presence of multiple extrapulmonary sarcoid localizations and increased 24-h urinary excretion of calcium were associated with significantly higher chitotriosidase activity (*p* < 0.005). Patients with remitted or minimal disease had lower values of chitotriosidase than patients with persistent disease. At follow-up, patients who required an increase in steroid dose showed an increase in its activity.

**Conclusions:**

Chitotriosidase is a reliable biomarker of sarcoidosis. It is increased in patients with sarcoidosis correlating with disease activity, severity and multiorgan dissemination. Steroid therapy tended to reduce chitotriosidase expression, however it responded in cases of disease relapse.

## Background

Sarcoidosis is a multi-organ granulomatous disease associated with abnormal T-lymphocyte and alveolar macrophage activation and migration into affected organs [1]. Since clinical course ranges from spontaneous recovery to severe deterioration [2], specific biomarkers would be useful to predict clinical outcome and guide therapeutic management [3].

Chitotriosidase is a chitinase involved in defense against chitin-containing pathogens [4]. Recent studies showed that the immunomodulatory effects of chitotriosidase go beyond innate immunity to involve macrophage maturation and differentiation, non-chitin antigen presentation and direct stimulation of many cytokines, such as IL-8 and TGF-β [5–9]. The enzyme has been found elevated in serum and bronchoalveolar lavage (BAL) of patients with sarcoidosis than in patients with other interstitial lung diseases, pulmonary tuberculosis and healthy controls [10, 11]. In sarcoidosis patients, chitotriosidase showed higher sensitivity and specificity than other biomarkers, including angiotensin converting enzyme (ACE), lysozyme and soluble IL-2 receptor [11, 12]. It has been found increased in active sarcoidosis patients [11] and showed to predict clinical course, steroid responsiveness and relapse of the disease [13].

The aim of the present study was to determine serum chitotriosidase activity in a large cohort of sarcoidosis patients in order to test its utility for identifying clinical phenotypes, different radiological lung involvement, extrapulmonary organ involvement, and evaluating its reliability in patients on steroid therapy.

## Materials and methods

### Study population and study design

#### Population

Between September 2015 and October 2017, serum chitotriosidase activity was assayed in 694 consecutive sarcoidosis patients (males 39%, age 55.8 ± 12.8 years) monitored at the Regional Referral Centre for Sarcoidosis and other Interstitial Lung Diseases, Siena, Italy. No quantifiable chitotriosidase activity (< 10 nmol/ml/h) was detected in 25 patients (3.6%), these patients were suspected with CHIT1 polymorphism, but they were included into analysis since no confirmatory genetic analysis was available. Healthy volunteers control group (*n* = 101, 34 males, mean age 52.2 ± 17.2 years; 73 non-smokers, 8 current smokers) without history of respiratory diseases and not receiving any therapy was also enrolled.

#### Diagnosis and measurements

Sarcoidosis was diagnosed according to international ATS/ERS/WASOG criteria [1]. All patients underwent complete physical evaluation and pulmonary function tests (PFT) including diffusing capacity of the lung for carbon monoxide (DLCO) and determination of serum concentrations of ACE and lysozyme. Combined physiological index (CPI) was calculated according to Walsh et al. [14] Extra-pulmonary sarcoid involvement (liver, spleen, chest and abdominal lymph nodes, skin, joints, heart, central nervous system) was assessed by specific diagnostic methods. Calcium metabolism was checked by 24-h urinary calcium excretion the same day as serum sampling.

#### Radiological evaluation

Chest x-ray alterations were classified according Scadding [15]. A high resolution CT (HRCT) scan of the chest was available for 228 patients. On the basis of the CT findings, patients were classified as having: 1) no lung or lymph node involvement; 2) parenchymal micronodules with or without lymph node enlargement; 3) parenchymal micro- and macro-nodules associated or otherwise with consolidation; 4) lung fibrosis, characterized by reticular abnormalities with or without traction bronchiectasis.

#### Patient stratification

According to therapy, patients were divided into two groups: *steroid-free* (*n* = 362) and *steroid* (*n* = 307). The latter included patients on steroids or other specific medications for sarcoidosis. Sarcoid patients were also classified according to the clinical phenotypes proposed by GenPhenResA [16] and, those with a follow-up of at least 5 years, with the Clinical Outcome Scale (COS) [17].

#### Follow-up

Clinical assessment and chitotriosidase assay was repeated at follow-up in a subgroup of patients (*n* = 416). Patients were classified at follow-up as “improved”, “stable” or “worse” according the physicians’ decisions on therapy (patients whose therapy was reduced or suspended were considered to have improved, patients whose therapy was maintained unchanged were considered stable, and patients whose therapy was increased (steroid dose or new drug added) were considered to be worse). All data was collected retrospectively.

### Chitotriosidase assay

Chitotriosidase activity was determined by a fluorimetric method using 22 μM 4-methylumbelliferryl β D-NNN-triacetylchitotriosidase (Sigma Chemical Co.) in citrate-phosphate buffer, pH 5.2; 100 μl substrate was incubated for 1 h at 37°C and the reaction was stopped with 1.4 ml 0.1 M glycine-NaOH buffer, pH 10.8, as previously described [10, 11]. Fluorescence was read at 450 nm with a Perkin Elmer LS40 fluorimeter (excitation wavelength 365 nm). Chitotriosidase activity in serum was expressed in nmol/ml/h.

### Statistical analysis

Data was expressed as mean ± standard deviation (M ± SD). Comparisons between groups were performed by t-test and one-way ANOVA with significance set at *p* ≤ 0.05. The Pearson test was used for correlation analysis. Contingency tables were analysed by Fisher’s exact test and Chi-square test. Outcome analysis was performed using the Mantel Cox test. Statistical analysis and graphic representations of the data were obtained using GraphPad Prism Version 5.0 software for Windows, while ROC curves were plotted using SPSS Statistics 20.

## Results

### Clinical, radiological and functional parameters

Demographic data, baseline pulmonary function test values and serum chitotriosidase, ACE and lysozyme activities of patients with sarcoidosis (*n*=694) and controls (*n*=101) are reported in Table 1. No significant differences in sex, age and smoking status were found between sarcoidosis patients and controls. On average, no significant impairment of PFT parameters, including DLCO, was found in our cohort of patients. Table 2 shows clinical, functional and laboratory data, as well as radiological staging and extra-pulmonary localizations of our sarcoidosis cohort.

**Table 1:** Demographic features, smoking status and biomarker assessment in the sarcoidosis cohort and healthy controls

	Sarcoidosis population	Controls	*p*-value
N°	694	101	
Male (%)	270 (39)	34 (33)	0.1310
Age (years)	55.8 ± 12.8	52.2 ± 17.2	0.2065
Smoking history (pack/year)	4.4 ± 9.7	5.1 ± 11.6	0.3457
• Current (%)	49 (7)	10 (10)	0.1199
• Former (%)	225 (32.4)	25 (25)	0.1199
• Never (%)	420 (60.5)	66 (66)	0.1199
Biomarkers determination (basal sampling)			
• Chitotriosidase nmol/ml/h	175.4 ± 89.4	34.2 ± 13.8	< 0.0001
• ACE UI/ml	48.5 ± 26.7	34.3 ± 21.8	0.0014
• Lysozyme mg/dl	5.4 ± 2.3	4.6 ± 2.1	0.0524

**Table 2:** Demographic data, smoking, comorbidities and functional, radiological and clinical parameters of the sarcoidosis cohort, divided into steroid-free and treated

	Sarcoidosis steroid-free	Sarcoidosis on therapy	*p*-value
N°	370	324	
Male (%)	160 (43)	128 (39.5)	0.4012
Age (years)	55.6 ± 14.9	54.2 ± 13.4	0.5978
Smoking history (pack/year)	4.8 ± 10	5.1 ± 10.4	0.1849
• Current (%)	18 (4.8)	19 (5.8)	0.4012
• Former (%)	117 (31.6)	79 (24.3)	0.4012
• Never (%)	235 (63.5)	226 (69.7)	0.4012
Biomarkers determination (basal sampling)			
• Chitotriosidase nmol/ml/h	180.1 ± 99.2	168.2 ± 118.2	0.3210
• ACE UI/ml	49.3 ± 24.2	48.5 ± 24.8	0.5031
• Lysozyme mg/dl	5.8 ± 1.8	5.4 ± 2.4	0.8265
Comorbidities	228 (61.2%)	199 (61.4%)	0.7887
• Arterial Hypertension (%)	93 (25.1)	67 (20.4)	0.1624
• Diabetes Mellitus (%)	21 (5.6)	27 (8.3)	0.0578
• Osteopenia/Osteoporosis (%)	112 (30.2)	110 (33.9)	0.1875
• Thyroid disorder (%)	29 (7.8)	25 (7.7)	0.9410
• Psychiatric disorder (%)	14 (3.7)	18 (5.5)	0.2178
• GERD/Hiatal ernia (%)	32 (8.6)	31 (9.5)	0.5264
• Other (%)	29 (7.8)	33 (10.1)	0.1765
PFTs			
• FVC l (%)	3.6 ± 1.1 (105.7 ± 18.2)	3.5 ± 1.1 (104.2 ± 18.4)	0.2455
• FEV1 l (%)	2.8 ± 0.9 (98.5 ± 19)	2.7 ± 1 (95.8 ± 19)	0.0945
• FEV1/FVC	76.1 ± 7.8	75.8 ± 8.7	0.6210
• TLC l (%)	6.3 ± 1.6 (110.8 ± 18.8)	6.1 ± 1.4 (109.1 ± 16.9)	0.3401
• DLCO %	83.1 ± 15.6	77.7 ± 16.9	0.002
• KCO %	92.7 ± 15.8	89.6 ± 15.8	0.0089
Radiological assessment (Scadding)			
• Stage 0 (%)	177 (47.8)	116 (35)	<0.0001
• Stage 1 (%)	45 (12.1)	28 (9)	<0.0001
• Stage 2 (%)	56 (15.1)	67 (21)	< 0.0001
• Stage 3 (%)	81 (21.8)	83 (24)	<0.0001
• Stage 4 (%)	11 (2.9)	30 (10)	<0.0001
Clinical assessment			
• No symptoms (%)	145 (39.1)	98 (30.2)	0.0123
• Cough (%)	132 (35.6)	110 (33.9)	0.8812
• Dyspnea (%)	155 (41.8)	133 (41)	0.8120
• Asthenia (%)	118 (31.8)	119 (36.7)	0.1125
• Arthtralgia (%)	71 (19.1)	76 (23.4)	0.1198
Localizations of disease			
• Isolated pulmonary	284 (76.7)	143 (44.1%)	<0.0001
• Lung + extrapulmonary	55 (14.8%)	111 (34.2%)	<0.0001
• Extrapulmonary only	31 (8.3%)	70 (21.6%)	<0.0001
Clinical phenotypes			
• Abdominal (%)	14 (3.7)	16 (4.9)	<0.0001
• OCCC (%)	12 (3.2)	20 (6.1)	<0.0001
• Muscoloskeletal-cutaneous (%)	35 (9.4)	78 (24)	<0.0001
• Isolated pulmonary (%)	284 (76.7)	143 (44.1)	<0.0001
• Extrapulmonary (%)	25 (6.7)	67 (20.6)	<0.0001
Lab parameters			
• 24 h urine calcium mg/dl	177.1 ± 136.8	222.6 ± 139.9	0.0289
COS classification	247	309	
• 1 (%)	49 (19.8)	0	<0.0001
• 2 (%)	50 (20.2)	0	<0.0001
• 3 (%)	33 (13.3)	0	<0.0001
• 4 (%)	29 (11.7)	0	<0.0001
• 5 (%)	31 (12.5)	0	<0.0001
• 6 (%)	55 (22.2)	10 (3.2)	<0.0001
• 7 (%)	0	112 (36.2)	<0.0001
• 8 (%)	0	151 (48.8)	<0.0001
• 9 (%)	0	36 (11.6)	<0.0001

*PFT* pulmonary function test, *COS* clinical outcome status and biomarker assessment in steroid-free and treated sarcoidosis patients, *GERD* gastro-esophageal reflux disease

### Chitotriosidase assay

**Fig. 1:**
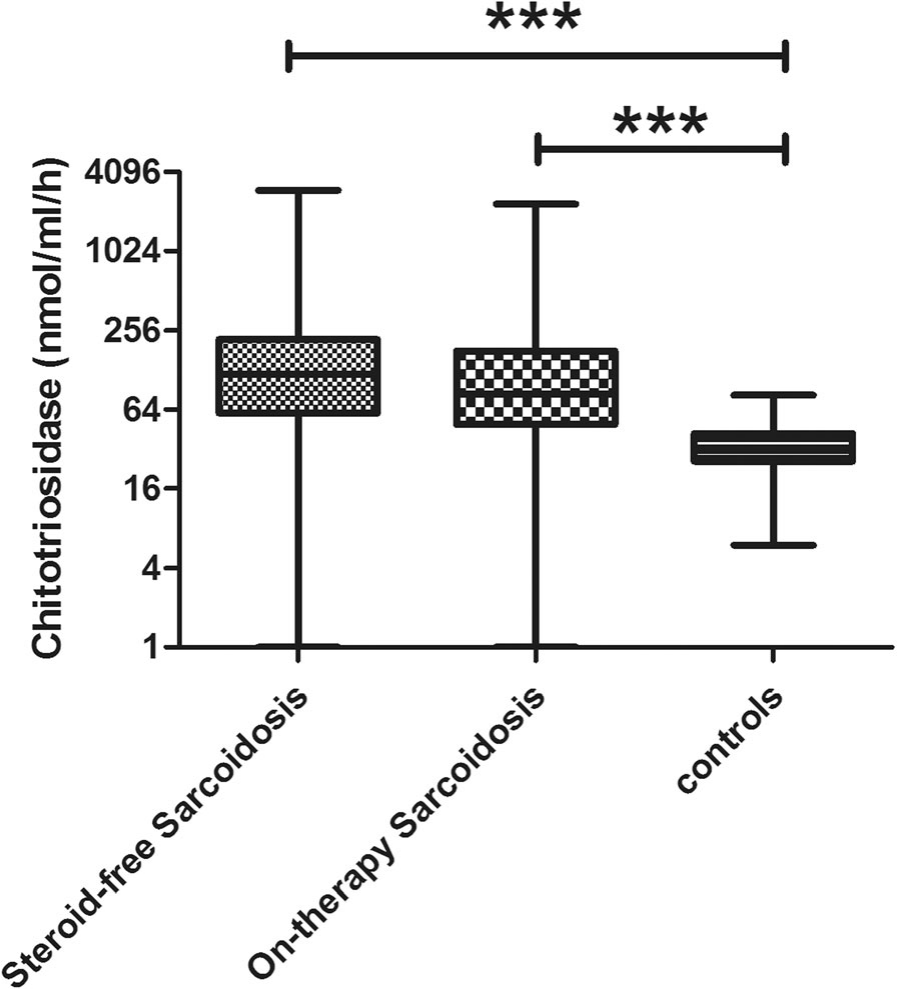
Comparison of chitotriosidase activity in steroid-free and treated sarcoidosis patients and healthy controls. Data expressed as mean ± standard deviation. ***: *p* < 0.0001. One-way ANOVA

Chitotriosidase activity was significantly higher in sarcoidosis patients than in healthy controls (t=5.490, *p*<0.0001). It was significantly higher in both steroid-free and treated groups of patients than in healthy controls (180.1 ± 99.2 and 168.2 ± 118.2 nmol/ml/h vs. 34.2 ± 13.8 nmol/ml/h, t=5.588 and t=5.492, respectively; *p*<0.0001 for both) (Fig. 1). The same was true for ACE levels (49.3 ± 24.2 U/l and 48.5 ± 24.8 U/l vs. 34.3 ± 21.8 U/l, t=3.344, *p*=0.0004 and t=2.883 *p*=0.004, respectively), whereas lysozyme levels were only significantly higher in steroid-free patients than healthy controls (5.8 ± 1.8 and 5.4 ± 2.4 mg/l vs. 4.6 ± 2.1;, t=1.998, *p*=0.046 and t=1.808 *p*=0.0721, respectively).

**Fig. 2:**
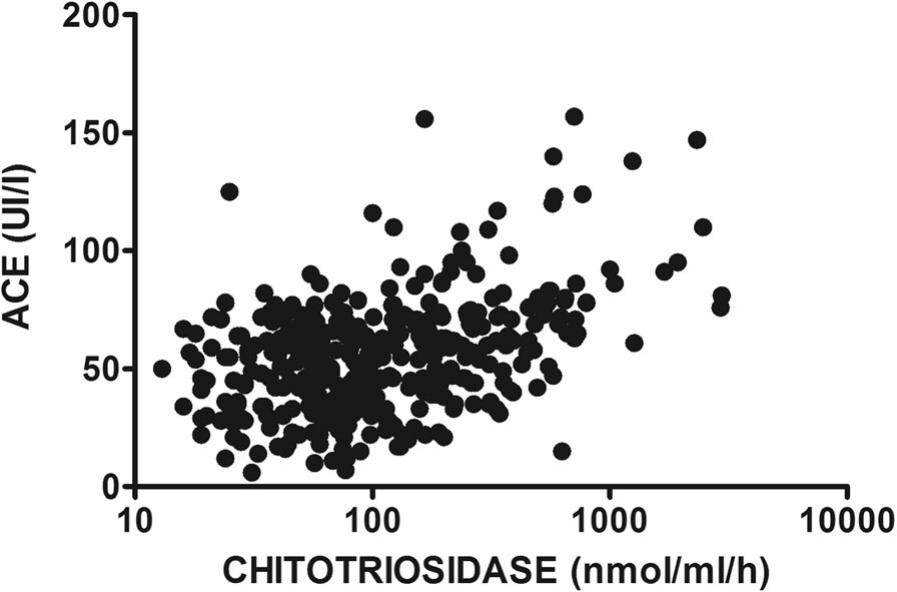
Correlation between chitotriosidase activity and ACE levels in the sarcoidosis cohort (*n* = 669) (*R* = 0.3513, *p* < 0.0001). Pearson’s correlation coefficient

In sarcoidosis patients, chitotriosidase activity and ACE concentrations were directly correlated (*r* = 0.34, *p*<0.0001) (Fig. 2). No significant differences were found in chitotriosidase activity according to sex, smoking status, age and comorbidities. Regarding specific symptoms, exertional dyspnea (t=2.714; *p*=0.0068) and cough were associated with higher chitotriosidase activity, however the latter was only statistically significant in steroid-free patients (t=2.287; *p*=0.0281).

Pulmonary function parameters did not significantly correlate with chitotriodase activity; however, stratifying patients according to specific patterns of alterations at PFTs (obstructive, restrictive or mixed respiratory defect), we observed that restrictive patients and those with reduced DLCO (TLC below the 5th percentile of the predicted value or DLCO < 75% of predicted) showed higher chitotriosidase activity (204 ± 305 vs 157 ± 242 nmol/ml/h, t=2.180, *p*=0.0296 and 310.8 ± 188 vs 162 ± 174 nmol/ml/h, t=2.682, *p*=0.0076, respectively). Chitotriosidase showed a significant correlation with CPI index as well (*r*=0.09434, *p*=0.0199).

Second line therapy approach included the use of methotrexate in 42 patients as steroid-sparing agent (27 patients) or as steroid resistant second-line treatment (15 patients): no differences of chitrotriosidase expression were found among these subgroups.

### Chitotriosidase and radiological features

Chitotriosidase activity was higher in patients with chest X-ray stages 1, 2, 3 and 4 than in those with stage 0 (*p*<0.0001).

High resolution CT evidence of lung fibrosis with reticular abnormalities and traction bronchiectasis (group 4) (*n*=12) was associated with significantly higher chitotriosidase activity than in patients with no evidence of lung involvement (q=4.654; *p*<0.001) or with only parenchymal micronodules and lymph node enlargement (q=4.286; *p*<0.01) or with parenchymal micro- and macro-nodules associated with consolidation (q=5.828; *p*<0.0001). Patients with HRCT evidence of lung fibrosis showed significant impairment of FVC, FEV1, DLCO and KCO compared to other patients and more frequently reported dyspnea; whereas arthralgia was more common in group 3 (parenchymal micro- and macro-nodules with consolidation) and group 4. Patients with lymph node enlargement and parenchymal micronodules (group 2) more frequently experienced an acute or subacute clinical onset of the disease (Table 3).

**Table 3:** Demographic, clinical and functional data and biomarker assessment of sarcoidosis patients in relation to High resolution computed tomography (HRCT) evidence of involvement

	No lung involvement	Lymph node + micronodular	Macronodular and consolidation	Lung fibrosis	*P*-value
N° (%)	27 (11.8)	127 (55.9)	62 (27.1)	12 (5.6)	
Male (%)	10 (39)	59 (46)	26 (42)	2 (16)	0.2063
Age yrs	57.6 ± 10.1	55.1 ± 12.7	57.3 ± 12.2	53.2 ± 12.7	0.5185
Smoking history (pack/year)	7.2 ± 11.7	3.8 ± 9	3.7 ± 9.1	8.2 ± 13.4	0.4052
• Current (%)	3 (11)	7 (5)	3 (5)	0	0.5357
• Former (%)	8 (29)	37 (29)	24 (38)	3 (25)	0.5357
• Never (%)	16 (59)	82 (65)	35 (56)	9 (75)	0.5357
Biomarkers determination (basal sampling)					
• Chitotriosidase nmol/ml/h	196.7 ± 150.9	284.2 ± 355.2	132.3 ± 119.7	666.3 ± 311	0.0005
• ACE UI/ml	51.4 ± 22.3	63.7 ± 31.3	50.1 ± 17.9	61.6 ± 26.1	0.0047
• Lysozyme mg/dl	4.5 ± 1.7	5.9 ± 2.2	5.1 ± 1.8	6.5 ± 2.4	0.0033
PFTs					
• FVC l (%)	3.6 ± 0.9 (107 ± 18)	3.4 ± 1.1 (100 ± 19)	3.5 ± 1.1 (108 ± 15.3)	2.7 ± 1.1 (79.3 ± 15.8)	<0.0001
• FEV1 l (%)	2.8 ± 0.8 (100.7 ± 18.7)	2.6 ± 0.9 (93.3 ± 19.8)	2.6 ± 0.9 (94 ± 15.6)	2.1 ± 0.8 (74.5 ± 17.3)	0.0013
• FEV1/FVC	77.2 ± 5.8	76.2 ± 8.6	75.1 ± 5	78.1 ± 8.2	0.3125
• TLC l (%)	6.2 ± 1.3 (109 ± 15.8)	6 ± 1.5 (105 ± 16.7)	6.2 ± 1.4 (115.4 ± 18.5)	4.6 ± 1.6 (91.5 ± 17.2)	0.0575
• DLCO %	85.3 ± 16.1	77.5 ± 14.9	79.5 ± 17.6	50.4 ± 16.2	<0.0001
• KCO %	96.3 ± 13.5	91.6 ± 19.3	89.2 ± 15.6	74.7 ± 17.8	0.0026
Clinical assessment					
• No symptoms (%)	18 (66)	44 (35)	17 (27)	2 (16)	0.0018
• Cough (%)	3 (11)	36 (28)	16 (26)	4 (33)	0.2981
• Dyspnea (%)	4 (15)	54 (43)	21 (34)	8 (66)	0.0074
• Asthenia (%)	4 (15)	39 (31)	16 (26)	5 (41)	0.2497
• Arthtralgia (%)	2 (7)	32 (25)	25 (40)	4 (33)	0.0118
Clinical onset					
• Asymptomatic (%)	24 (89)	72 (57)	47 (76)	10 (83)	0.0017
• Lofgren syndrome (%)	1 (3)	8 (6)	4 (6)	0	0.7808
Clinical phenotypes					
• Abdominal (%)	1 (3)	14 (11)	1 (1.6)	2 (16)	0.5170
• OCCC (%)	2 (7)	7 (5)	3 (5)	0	0.5170
• Muscoloskeletal-cutaneous (%)	3 (11)	16 (12.6)	9 (14)	1 (8)	0.5170
• Isolated pulmonary (%)	21 (77)	88 (69.2)	48 (77)	9 (75)	0.5170
• Extrapulmonary (%)	0	2 (1.5)	1 (1.6)	0	
COS classification	19	95	51	10	
• 1 (%)	4 (21)	1 (1)	0	0	0.0002
• 2 (%)	3 (16)	3 (3)	0	0	0.0002
• 3 (%)	2 (10)	3 (3)	0	0	0.0002
• 4 (%)	2 (10)	3 (3)	3 (6)	0	0.0002
• 5 (%)	1 (5)	4 (4)	3 (6)	0	0.0002
• 6 (%)	1 (5)	9 (9)	9 (17)	2 (20)	0.0002
• 7 (%)	4 (21)	26 (21)	10 (19)	1 (10)	0.0002
• 8 (%)	2 (10)	36 (36)	22 (43)	5 (50)	0.0002
• 9 (%)	0	11 (11)	4 (8)	2 (20)	0.0002

*OCCC* ocular-cardiac-cutaneous-central nervous system

### Chitotriosidase activity in relation to extrapulmonary localizations and GenPhenResA phenotypes

**Fig. 3:**
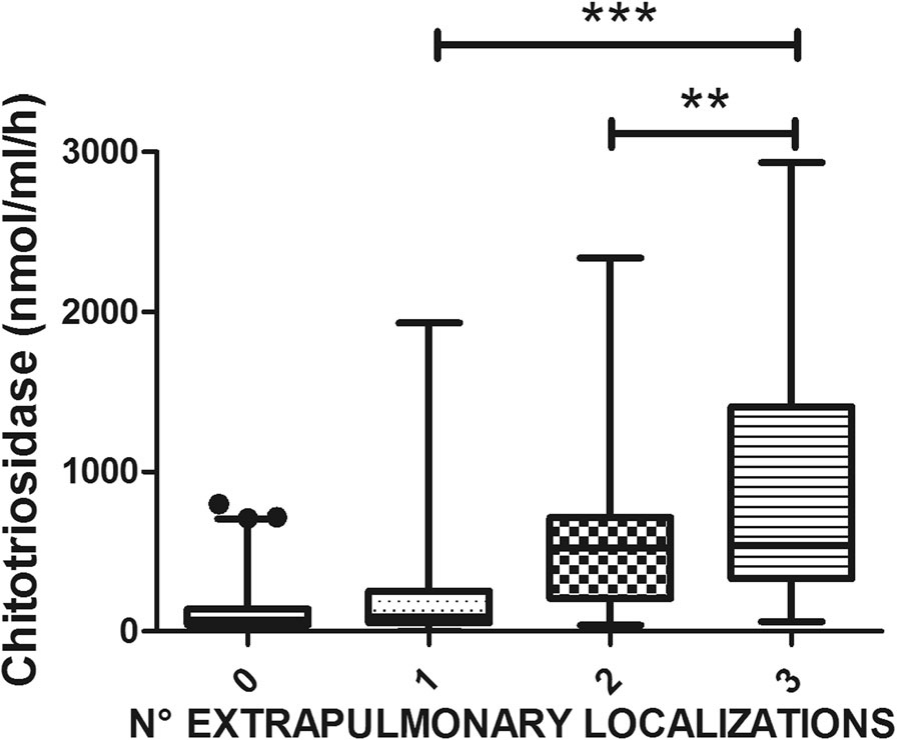
Comparison of Chitotriosidase activity in Sarcoidosis patients, classified on the basis of the number of extrapulmonary localizations. Data expressed as mean ± standard deviation. **: *p* < 0.01; ***: *p* < 0.0001. One-way ANOVA

The number of patients with at least one extrapulmonary localization of sarcoidosis was 267 out of 694 (38.4%). They showed significantly higher chitotriosidase activity than patients with isolated pulmonary disease (t=5.257, *p*<0.0001). Chitotriosidase activity tended to be progressively higher in patients with multiple organ involvement. In particular, patients with three or more disease localizations reported significantly higher chitotriosidase activity than those with one or two localizations (q=10.79, *p*<0.0001 and q=5.05, *p*<0.001, respectively) (Fig. 3). Extrapulmonary disease was associated with significantly increased chitotriosidase activity, regardless pulmonary involvement (F=10.51; *p*<0.0001). Chitotriosidase in patients with lung involvement only was 144.1 ± 188.2 nmol/ml/h; in patients with lung and extrapulmonary was 239.4 ± 398.6 nmol/ml/h; in patients with extrapulmonary only was 249.5 ± 393.1 nmol/ml/h.

A correlation between chitotriosidase and 24h-urinary calcium excretion was observed in both steroid-free and steroid subgroups (*r*=0.4201, *p*<0.0001; *r*=0.3528; *p*=0.0004).

**Fig. 4:**
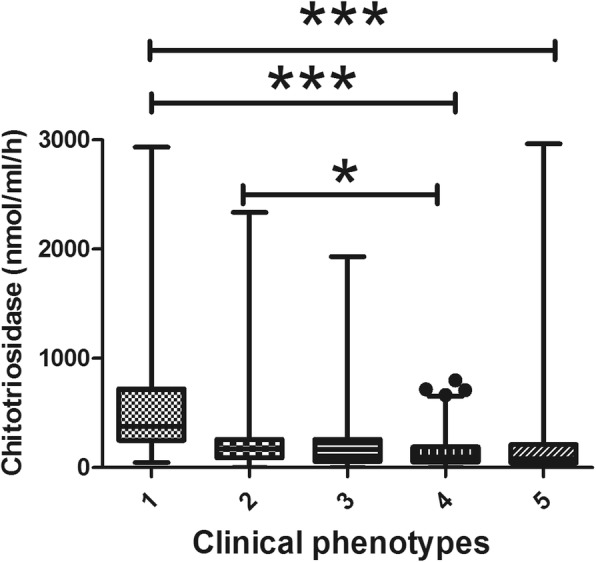
Comparison of chitotriosidase activity in different GenPhenResA phenotypes. 1: abdominal; 2: ocular-cardiac-cutaneous-central nervous system; 3: muscoloskeletal-cutaneous; 4: pulmonary; 5: extrapulmonary. Data expressed as mean ± standard deviation. ***: *p* < 0.0001; *: *p* < 0.05. One-way ANOVA

According GenPhenResA phenotypes [16], patients with abdominal organ involvement had higher chitotriosidase activity than other groups (q=8.155, *p*<0.0001 vs ocular-cardiac-cutaneous-central-nervous-system; q=11.32, *p*<0.0001 vs musculoskeletal-cutaneous; q=14.97, *p*<0.0001 vs isolated pulmonary disease; q=11.49, *p*<0.0001 vs extrapulmonary disease), while ocular–cardiac–cutaneous–central-nervous-system (OCCC) localization showed higher chitotriosidase activity than isolated pulmonary involvement (q=4.494; *p*<0.05) (Fig. 4).

### Chitotriosidase activity in relation to COS classification

**Fig. 5:**
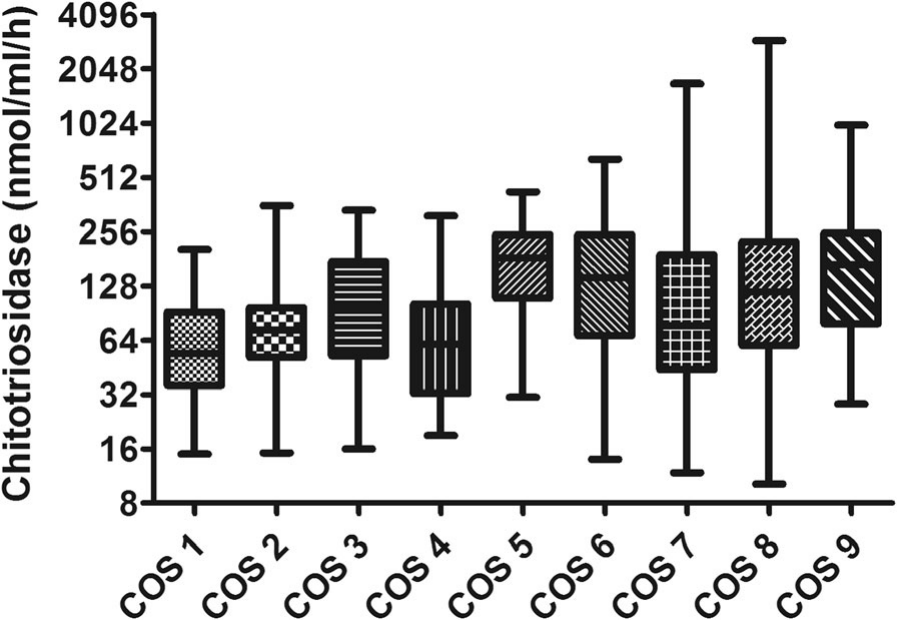
Comparison of chitotriosidase activity in relation to COS classification. Significant differences emerged between COS 1 and COS 5–6–8-9 patients (*p* < 0.0001 in all cases), COS 2 and COS 5–6-9 patients (*p* < 0.05, *p* < 0.05 and *p* < 0.0001, respectively), COS 4 and COS 5–6–8-9 patients (*p* < 0.001, *p* < 0.001, *p* < 0.05 and *p* < 0.0001, respectively), COS 5 and COS 7 patients (*p* < 0.05) and COS 7 and COS 9 patients (*p* < 0.001). Data expressed as mean ± standard deviation. One-way ANOVA

The number of patients that could be classified by COS [17] was 556: 99 were in remission (COS 1-2), 62 reported minimal disease (COS 3-4) and 395 showed persistent disease (COS 5 to 9). Statistically significant differences in chitotriosidase activity were found between COS-1 and COS-5-6-8-9 (*p*<0.001), COS-2 and COS-5-9 (*p*<0.01), COS-4 and COS-5-6-8-9 (*p*<0.01, *p*<0.05, *p*<0.05 and *p*<0.01, respectively) and COS-7 and COS-9 (*p*<0.05). Clinically persistent disease was associated with significantly higher chitotriosidase activity than minimal or remitted disease (t=3.824, *p*=0.0001) (Fig. 5).

**Fig. 6:**
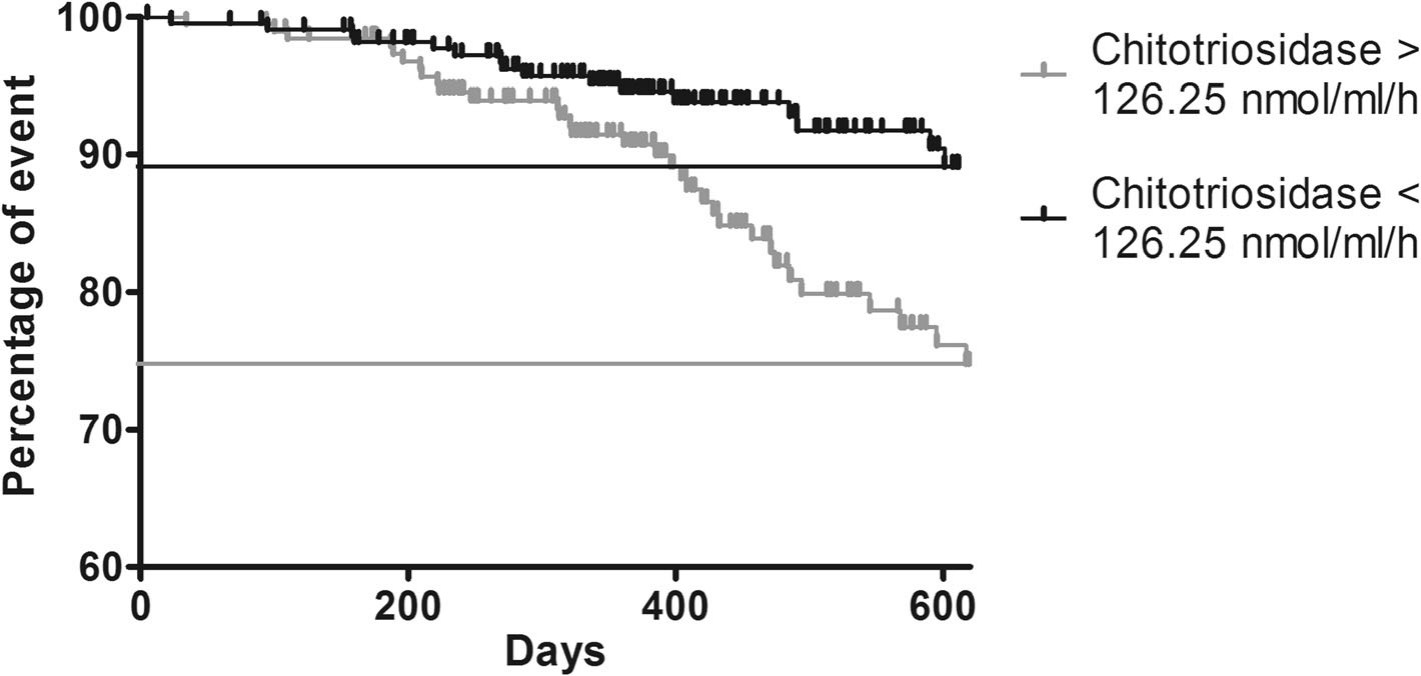
Log-rank test comparing outcome (increase in daily dose of steroid) in sarcoidosis patients with basal chitotriosidase above or below the cut-off of 126 nmol/ml/h. (Chi-square = 10.396; *p* = 0.001). Long rank test

Patients with chitotriosidase activity exceeding 126.25 nmol/ml/h more frequently showed persistent disease as defined by COS classification (sensitivity 57%, specificity 72%) and more frequently needed a higher daily dose of steroid (48 vs 26 events, prevalence: 24.8% and 11%, respectively) (Fig. 6).

### Chitotriosidase activity at follow-up

Clinical assessment and chitotriosidase determination was available at follow-up in 416 patients (175 males, age 56 ± 13.8 years) (baseline to follow-up interval: 414 ± 265 days).

**Fig. 7:**
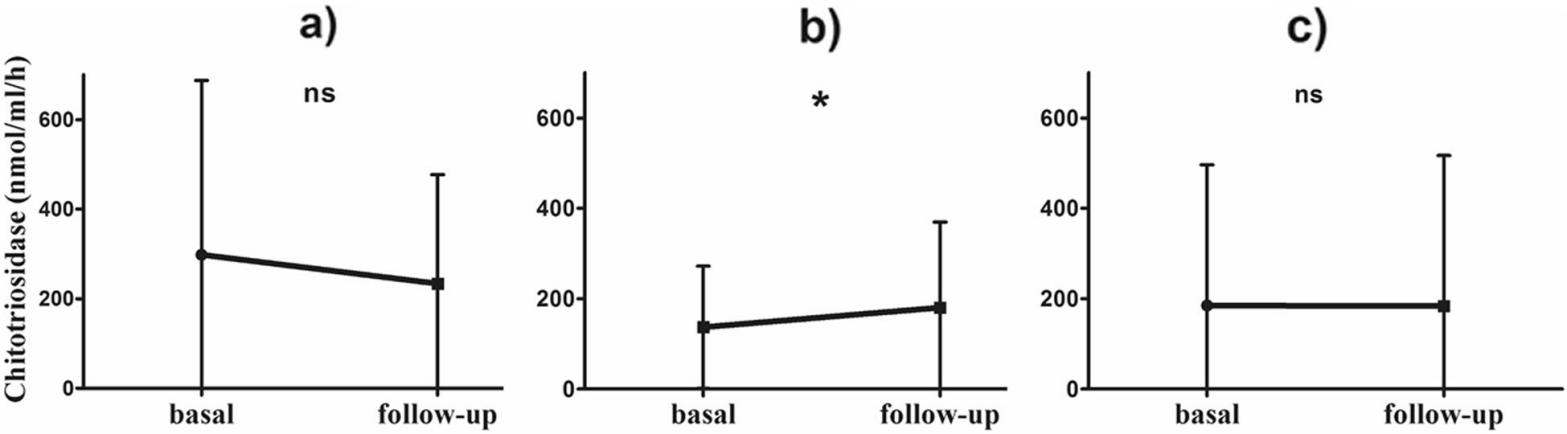
Line plots of Chitotriosidase activity at basal and follow-up sampling: **a** therapy modification, *improved*; **b** therapy modification, *worsened*; **c** therapy modification, *stable*. *: 0.0012. Paired t test

When physicians decided to change steroid dose or otherwise modify therapy, a significant increase in chitotriosidase activity was observed in "worse" patients (*n*=215) (Table 4), whereas "improved" (*n*=47) and "stable" (*n*=164) patients did not show any significant change (Fig. 7).

**Table 4:** Changes in chitotriosidase activity between basal and follow-up sampling, in relation to subjective symptoms and therapy adjustments during the observation period

	Δ Chitotriosidase (nmol/ml/h)	*p*-value
Therapy modification		
• Improved (*n* = 47)	−64.5 ± 118.1	0.2045
• Stable (*n* = 164)	−1.3 ± 214.4	0.9389
• Worsened (*n* = 215)	44.1 ± 131.4	0.0012

## Discussion

The present study offers new insights into the clinical utility of chitotriosidase as a biomarker in patients with sarcoidosis. Sarcoidosis is a complex, pleiotropic disease with many phenotypes and many clinical classifications have been proposed [18]. Numerous biomarkers have been proposed over time in sarcoidosis, but none of these have been universally recognized in clinical practice [19–21]. Ideal characteristics of candidate biomarkers should be highly specific and sensitive for the diagnosis, but more importantly, in sarcoidosis patients, from a prognostic point of view, to discriminate among different phenotypes and states of the diseases predicting remission or progression [22]. In the present study we aimed to test chitotriosidase utility for identifying clinical phenotypes, different radiological lung involvement, extrapulmonary organ involvement, and for evaluating its reliability in a large cohort of sarcoidosis patients with and without steroid therapy.

Chitotriosidase is a chitinase involved in defense against chitin-containing pathogens [4]. This enzyme, implicated in innate immunity, macrophage maturation and differentiation, has been found elevated in serum and bronchoalveolar lavage (BAL) of patients with sarcoidosis and has been proposed some years ago as prognostic biomarker by our group of research [11]. It showed higher sensitivity and specificity than other biomarkers [23–26], including angiotensin converting enzyme (ACE), lysozyme and soluble IL-2 receptor [11, 12] and it has been found increased in active sarcoidosis patients [11] showing to predict clinical course, steroid responsiveness and relapse of the disease [13].

In the present large case series (*n* = 694), chitotriosidase activity proved to be a reliable biomarker of sarcoidosis; it was significantly increased in patients with sarcoidosis than in healthy controls and it correlated with respiratory symptoms (exertional dyspnea and cough) and with serum ACE concentration. It also proved to correlate with disease severity, expressed as severe progressive pulmonary and extrapulmonary involvement, to be able to detect disease relapse and to identify patients requiring escalation of therapy.

Chitotriosidase correlated with radiological findings in our cohort: patients with chest X-ray stages different from 0 showed significantly higher concentrations. In the present study, we firstly investigated correlations between chitotriosidase activity and different CT patterns of presentation. Severe pulmonary involvement, indicated by HRCT by evidence of lung fibrosis with reticular abnormalities and traction bronchiectasis, was associated with a significant increase in chitotriosidase activity. In sarcoidosis patients, fibrosis is a result of persistent and uncontrolled disease [27] and chitotriosidase may reflect persistent granuloma activity. We also explored Chitotriosidase activity in relation the composite physiological index (CPI), proposed by the Brompton group of research, that showed to identify patients at high clinical risk correlating with radiological extent of fibrosis [14] and we found a slightly significant correlation with chitotriosidase activity. In our cohort strength of such correlation is rather weak, even if significant (*r* = 0.09434, *p* = 0.0199); probably it is underestimated by the overwhelming percentage of patients with normal PFTs. Further studies are needed to better understand values of CPI in combination with chitotriosidase in sarcoidosis, but indirectly suggests the association of chitotriosidase expression with fibrosis development. Overexpression of chitotiosidase has been previously associated with abnormal production of TGF-beta, leading to initiation and perpetuation of interstitial lung disease in systemic sclerosis [9] and its expression, and of other chitinases (i.e. YKL-40), has been associated to airway remodelling in severe asthma [28, 29]. Moreover, in BAL from patients with sarcoidosis, chitotriosidase has been found increased in progressive than stable patients [11, 24]. These results suggest that this enzyme could be involved in fibrogenesis in diffuse lung diseases, expression of high activation of certain macrophage pathways responsible for remodeling and fibrosis development.

In our study, population was divided according to GenPhenReSa phenotypes classification [16], that have been proposed to make homogenous cohorts in biomedical studies, in five subgroups: 1) abdominal organ involvement; 2) ocular–cardiac–cutaneous–central; nervous system disease involvement; 3) musculoskeletal–cutaneous involvement; 4) pulmonary and intrathoracic lymph node involvement; 5) extrapulmonary involvement [15]. We did observe that patients with multiorgan sarcoid involvement (39% of our population), particularly with abdominal involvement, had higher chitotriosidase activity: patients with three or more extrapulmonary localizations showed higher chitotriosidase. In 2016, Popevic et al. failed to demonstrate higher chitotriosidase levels in patients with extrapulmonary sarcoidosis detected by PET-CT scan [23]. In the present study, the definition of extrapulmonary involvement was based on specific clinical, radiological and pathological investigations, including PET-CT scan in some cases. This approach, probably more complete and sensitive than PET-CT scan alone, might explain our findings. Patients with active and/or persistent disease showed increased activity of chitotriosidase. Definition of sarcoidosis activity is difficult and there is still no universal consensus. In the present study, we used the COS classification scale proposed some years ago by WASOG in which patients are classified in relation to need for therapy, disease persistence, relapse of symptoms and modification of therapy at follow-up [17]. It has been previously reported by us and others [10, 23] that chitotriosidase activity is expressed differently among COS stages. Even if we could not apply COS to all our population, but only to patients with 5 or more years of follow-up at time of chitotriosidase determination (*n* = 553), in the present study we confirmed this association: patients in remission or with minimal disease (COS stages 1 to 4) had lower chitotriosidase activities than patients with persistent disease (COS stages 5 to 9) and, among the latter, patients whose conditions deteriorated showed higher activities than stable patients.

Chitotriosidase expression showed to increase in patients with multiple organ involvement suggesting that its level correspond to the amount of active organ involvement reflecting the number of active granulomas harbored by a patient at a given time. In a previous paper from our group [24], we reported a significant discrepancy between serum and BAL expression of chitotriosidase suggesting the potential extrapulmonary production of this biomarker, however in that paper population was not such well characterized as in this present work. Anyway, extrapulmonary production of chitotriosidase is supported by other studies describing chitotriosidase as a reliable marker of macrophage activation in different organs and apparatus [30–32].

Chitotriosidase is mainly secreted by macrophages and is involved in maturation of monocytes into both M1 and M2 macrophages subtypes, even without the presence of chitin. How chitotriosidase is implicated in pathogenic mechanisms of sarcoidosis is unknown. The pathogenesis of sarcoidosis is complex and not yet completely understood; environmental, genetic and immunological factors demonstrated several interactions that lead to macrophage activation and granuloma formation. The innate immune system has also been shown to play a role in the triggering and maintenance of granulomatous inflammatory phenomena [33]. However, the events preventing dissolution of granulomas and maintaining inflammation, evolving toward fibrosis are not yet fully characterized [33] and specific studies to better understand chitotriosidase role in sarcoidosis mechanisms are lacking.

The chitotriosidase activity cut-off of 48.8 nmol/h/ml was proposed by our research group to distinguish sarcoid patients from healthy subjects with high sensitivity and specificity (89 and 93%, respectively) [10]. Chitotirosidase has been found overexpressed in sarcoidosis than patients with idiopathic pulmonary fibrosis, interstitial lung disease associated to systemic sclerosis and tubercolosis [24, 25], however increased levels have been also reported in COPD, asbestosis and lung cancer patients [26]. Diagnostic accuracy of chitotriosidase for sarcoidosis is still to be addressed, prospective studies evaluating its levels in patients with differential diagnosis with sarcoidosis are strongly needed.

In the present study, we identified a second cut-off to predict disease persistence among sarcoidosis: patients with chitotriosidase activity higher than 126 nmol/h/ml proved to have active disease requiring an increase in therapy. These observations confirm the prognostic value of this biomarker, which can identify patients most likely to have a persistent disease and therefore requiring particular attention. In fact, chitotriosidase analysis at follow-up showed that patients requiring an increase in steroid dosage showed higher chitotriosidase activity. In line with this observation, Harlander et al. reported a significant increase in chitotriosidase activity during relapse of sarcoidosis [13]. Unfortunately, due to retrospective nature of this study, we could not explore chitotriosidase in relation with quality of life as standardized specific questionnaires were not available for the majority of patients included in the study.

The main limitations of the present study are its retrospective and monocentric nature and the absence of a validation cohort. Moreover, the low number of patients with Löfgren syndrome prevented us to explore chitotriosidase in this particular subgroup of patients. However, this is the biggest study ever conducted on chitotriosidase in sarcoidosis and the results clearly show its potential utility as a clinical biomarker correlating with many clinical and radiological parameters. Prospective studies to evaluate its role in clinical decision-making are needed to definitely establish its role in sarcoidosis patients.

## Conclusion

The present study demonstrated that chitotriosidase activity is a reliable biomarker of sarcoidosis. Our results showed that it is correlated with disease activity, severity and multiorgan dissemination. Taken together, our findings support an intriguing hypothesis that chitotriosidase production, mainly by sarcoid macrophages [34], could reflects the number of active granulomas harbored by a patient at a given time. In favor of this speculation, we observed chitotriosidase activity differently expressed in patients with severe pulmonary involvement (presence of lung fibrosis at CT scan), with multi vs. single organ involvement (in particularly in patients with abdominal involvement and with three or more extrapulmonary localizations), with active vs. non-active disease and with remitted/minimal vs. persistent disease and in patients at high clinical risk (expressed by CPI index). Moreover, chitotriosidase expression reduces with steroid therapy and it proved able to detect disease relapse and to identify patients requiring escalation of therapy. Together with these positive clinical findings, chitotriosidase laboratory determination has been shown feasible, quick and accurate with reasonable costs [35].

Combined with clinical, radiological and physiological findings, chitotriosidase activity proved to be an excellent non-invasive prognostic biomarker for management of patient with sarcoidosis, with a cost-benefit ratio highly positive, that should be part of the regular follow-up of sarcoidosis patients in the daily clinical practice.

## Data Availability

databases and all relevant raw data can freely available on request.

## Abbreviations

ACE: Angiotensin converting enzyme
BAL: Bronchoalveolar lavage
COS: Clinical Outcome Scale
FEV1: Forced expiratory volume in 1 s
FVC: Forced vital capacity
HRCT: High resolution computed tomography
KCO: Diffusing capacity of the lung for carbon monoxide/alveolar volume
PFT: Pulmonary function tests
DLCO: Diffusing capacity of the lung for carbon monoxide
