# Correction to: Chitotriosidase: a biomarker of activity and severity in patients with sarcoidosis

**DOI:** 10.1186/s12931-020-1303-8

**Published:** 2020-01-29

**Authors:** David Bennett, Paolo Cameli, Nicola Lanzarone, Loredana Carobene, Nicola Bianchi, Annalisa Fui, Luigi Rizzi, Laura Bergantini, Giuseppe Cillis, Miriana d’Alessandro, Maria Antonietta Mazzei, Rosa Metella Refini, Piersante Sestini, Elena Bargagli, Paola Rottoli

**Affiliations:** 1 Respiratory Diseases and Lung Transplantation Unit, Azienda Ospedaliera Universitaria Senese, Siena, Italy; 2 Department of Medical and Surgical Sciences & Neurosciences, University of Siena, Siena, Italy; 3 Internal Medicine Unit “C. Frugoni”, Centre for Rare Diseases, University Hospital of Bari, Bari, Italy; 4 Diagnostic Imaging Unit, Azienda Ospedaliera Universitaria Senese, Siena, Italy

**Correction to: Respir Res (2020) 21:6**

**https://doi.org/10.1186/s12931-019-1263-z**

After publication of our article [1] the authors have notified us that one of the names has been incorrectly tagged.

- Original name tagging:

Given name: d’Alessandro

Family name: Miriana

- Correct name tagging:

Given name: Miriana

Family name: d’Alessandro

The original article has been corrected.
